# Risk factors for suicide in Hungary: a case-control study

**DOI:** 10.1186/1471-244X-9-45

**Published:** 2009-07-28

**Authors:** Kitty Almasi, Nora Belso, Navneet Kapur, Roger Webb, Jayne Cooper, Sarah Hadley, Michael Kerfoot, Graham Dunn, Peter Sotonyi, Zoltan Rihmer, Louis Appleby

**Affiliations:** 1 In and Outpatient Department of Psychiatry, No III, National Institute for Psychiatry and Neurology, Budapest, Hungary; 2 Centre for Suicide Prevention, University of Manchester, Manchester, M13 9PL, UK; 3 Biostatistics/Health Methodology Research Group, University of Manchester, Manchester, M13 9PL, UK; 4 Psychiatry Research Group, University of Manchester, Manchester, M13 9PL, UK; 5 Department of Forensic Medicine, Semmelweis Medical University, Budapest, Hungary; 6 Department of Psychiatry and Psychotherapy, Semmelweis Medical University, Budapest, Hungary

## Abstract

**Background:**

Hungary previously had one of the highest suicide rates in the world, but experienced major social and economic changes from 1990 onwards. We aimed to investigate the antecedents of suicide in Hungary. We hypothesised that suicide in Hungary would be associated with both risk factors for suicide as identified in Western studies, and experiences related to social and economic restructuring.

**Methods:**

We carried out a controlled psychological autopsy study. Informants for 194 cases (suicide deaths in Budapest and Pest County 2002–2004) and 194 controls were interviewed by clinicians using a detailed schedule.

**Results:**

Many of the demographic and clinical risk factors associated with suicide in other settings were also associated with suicide in Hungary; for example, being unmarried or having no current relationship, lack of other social contacts, low educational attainment, history of self-harm, current diagnosis of affective disorder (including bipolar disorder) or personality disorder, and experiencing a recent major adverse life event. A number of variables reflecting experiences since economic restructuring were also associated with suicide; for example, unemployment, concern over work propects, changes in living standards, practising religion. Just 20% of cases with evidence of depression at the time of death had received antidepressants.

**Conclusion:**

Suicide rates in Hungary are falling. Our study identified a number of risk factors related to individual-level demographic and clinical characteristics, and possibly recent societal change. Improved management of psychiatric disorder and self-harm may result in further reductions in suicide rates.

## Background

Suicide rates are elevated in large parts of Northern and Eastern Europe, for example the Baltic countries, Belarus, Croatia, Finland, Hungary, the Russian Federation, Slovenia and the Ukraine [1]. Some of the highest figures have been reported in Hungary [2]. Since the fall of Communist governments, many countries in Central and Eastern Europe have experienced major social, political, and economic upheaval. In Hungary since 1990, there have been significant increases in unemployment, poverty, alcohol misuse, and divorce [3]. At the same time healthcare systems in the region have undergone extensive changes [4], with increases in the provision of mental health services [2]. There has been an increase in religious and other freedoms. The current psychological autopsy study provided a unique opportunity to investigate the antecedents of suicide in this setting. We hypothesised that suicide in Hungary would be associated with:

1. Social, clinical and behavioural risk factors that have been reported as being associated with suicide in Western studies, such as social isolation, life events, severe mental illness, personality disorder, previous self-harm, and alcohol/drug misuse.

2. Experiences related to social and economic developments since 1990. These included both possible risk factors such as unemployment and socio-economic decline, and protective factors such as increased religious observance.

## Methods

### Setting

A population-based case series was identified through the Department of Forensic Medicine at Semmelweis Medical University. Post-mortem examinations are routinely carried out in this department on all those who die by suicide in the city of Budapest and the surrounding county of Pest (population 1.6 million and 0.5 million respectively). Within a few days of a death family members collect a death registration document from the Department of Forensic Medicine which allows them to proceed with funeral arrangements. Controls were identified through the general practitioners of the deceased.

### Subjects

Cases were individuals who died by suicide during a 24-month period (March 2002 – March 2004). In Hungary the police make the initial judgement that a suicide has occurred and this is confirmed on the basis of a post-mortem examination and toxicology. Open verdicts are rarely assigned and, as in previous Hungarian studies of suicide [5], were not included in the current study. Subjects were excluded if they lived outside Budapest or Pest County or if they lacked a suitable informant. However these exclusions were comparatively rare – approximately 7% of all individuals who died by suicide during 2002 and 2003 were from outside the study area and only 1% did not have an informant.

Informants (most commonly family members) were approached to take part in the study at the time they visited the Department of Forensic Medicine to collect documentation. The interviews were arranged for the same day or at a later date at the informant's convenience. They took place either at the Department of Forensic Medicine or in the informant's home. Permission was also sought to contact the deceased person's general practice and access health records. The general practitioners were identified through relatives, forensic files or the National Institute of General Practitioners in Hungary. Due to resource constraints and to assist with scheduling of the interviews it was not possible to recruit informants for all cases of suicide which occurred during the study period. We sought to recruit a representative sample by approaching informants on three of the five days per week that the Department was open (Monday, Wednesday, Thursday).

Controls were matched for age (within three years), gender, and general practice. For each individual who died by suicide, the general practitioner identified all individuals of the same gender who had a date of birth within three years of the case from the practice register. They then contacted the control subject with a date of birth closest to that of the case. Controls were contacted by telephone about the study in most instances and were asked to nominate a suitable informant for interview. In instances where the control subject felt unable to nominate another person, they acted as their own informant and underwent the interview themselves. As with the cases, permission was sought to access health records. When individuals refused to participate, the general practitioner contacted the control subject with the next closest date of birth.

After complete description of the study to all cases and controls, written informed consent was obtained. The study was approved by the Ethical Committee of Semmelweis Medical University, Budapest (Hungary) and the South Manchester Local Research Ethics Committee (England).

### Assessment

We used a psychological autopsy methodology [6]. The main sources of information were the informants. They were interviewed by experienced clinicians (KA, NB) using a semi-structured interview schedule based on those used in previous psychological autopsy studies [7,8]. Information on demographic and clinical characteristics, life events and difficulties [9], personality [10], and mental state was collected for both cases and controls. The mental state section consisted of items from the Hungarian version of the Mini Neuropsychiatric Interview (M.I.N.I. v5.1) [11-13], which can be used to generate DSM-IV diagnoses. The schedule also included items which reflected experiences since economic restructuring. As well as collecting information on current mental illnesses at the time of suicide, informants were also asked to indicate whether or not the subject had a psychiatric history prior to the current episode. Details of the suicide episode were collected for cases only. Information regarding the relationship to the deceased was collected for all informants.

The median number of days from death to informant interview was 5 days for the cases: IQR 3 days to 8 days, ranging from 0 days (that is day of death) to 260 days. The reference date in control interviews – the point of comparison for events prior to suicide – was the date of interview itself. To use the date of suicide, usually several months earlier, may have impaired recall among control informants. The median length of interviews with informants was one hour for the cases of suicide and 40 minutes for the controls.

Disagreements over diagnosis or personality assessments were resolved at consensus meetings of the research team. Information from the interviews was supplemented with data from the forensic medicine files. General practice case records were also examined for subjects where there was incomplete information (number of records inspected 53/194 for the cases and 38/194 for the controls).

### Statistical analyses

The intended sample size was 200 cases and 200 controls. This would have provided 85% power to detect a statistically significant difference in exposure prevalence of 25% in the suicide group and 12.5% in the control group (odds ratio = 2.3).

Conditional logistic regression was used to account for the matched design. Variables were grouped together in 4 domains (see Tables 1, 2 and 3): Socio-demographic factors; other background factors; clinical factors; and life events. To simplify analysis and interpretation, life events were classified in five categories [14]: interpersonal life events; work/education life events; health life events (relating to self or close others); financial life events; forensic or legal life events. Stata software [15] was used to calculate odds ratios and their 95% confidence intervals. Univariate analyses were carried out initially to investigate the association between individual factors and the risk of suicide. To avoid model instability problems we did not fit univariate models based on a value of less than 5 in one or more cells when cross-tabulated against the case-control binary variable [16] – (this criterion applies to all analyses presented in Tables 1, 2 and 4). Multivariate models were then generated within each domain using backwards elimination procedures; explanatory variables with less than 10 subjects in one or more cells were not fitted in these models (including the final model: Table 3). The predictors from each of the domain specific models were then fitted in a final multivariate model to identify risk factors independently associated with suicide. Explanatory variables were retained in the final model if the p-value was less than 0.05.

**Table 1:** Univariate conditional logistic regression models of socio-demographic and other background risk factors for suicide^a^

Risk factor	Cases n (%)	Controls n (%)	Odds Ratio (95% CI)	p-value
**Socio-demographic factors**				
Marital status:^b^				
(i) Single	60 (31.7)	49 (25.9)	3.29 (1.42–7.64)	0.006
(ii) Separated/divorced/widowed	63 (33.3)	33 (17.5)	3.46 (1.90–6.30)	< 0.001
Non-white ethnic origin	5 (2.6)	16 (8.2)	0.21 (0.06–0.75)	0.02
Living alone	55 (28.4)	49 (25.3)	1.18 (0.74–1.86)	0.49
No current relationship	84 (45.7)	46 (25.0)	3.53 (1.99–6.27)	< 0.001
Responsible for child aged < 18 yrs.	34 (17.8)	47 (24.6)	0.46 (0.22–0.94)	< 0.001
Not been out socially in last month	96 (52.7)	33 (18.1)	4.94 (2.89–8.45)	< 0.001
Practising a religion	30 (15.6)	67 (34.9)	0.31 (0.18–0.54)	< 0.001
No education beyond age 16 yrs.	46 (23.7)	15 (7.7)	5.43 (2.42–12.16)	< 0.001
Improved standard of living since 1990	6 (3.2)	29 (15.6)	0.08 (0.02–0.34)	= 0.001
Unemployed or long-term sick/disabled	33 (17.0)	6 (3.1)	7.75 (2.74–21.95)	< 0.001
Ever been unemployed since 1990^c^	52 (40.0)	27 (20.8)	2.67 (1.47–4.83)	< 0.001
Concerns over work prospects^c^	84 (68.9)	35 (28.7)	5.90 (3.02–11.53)	< 0.001

**Other background factors**				
Born prematurely	32 (20.6)	30 (19.4)	1.09 (0.62–1.91)	0.77
Suicide by 1^st ^degree relative	31 (16.4)	6 (3.2)	6.00 (2.33–15.46)	< 0.001
Adopted	12 (6.2)	6 (3.1)	2.00 (0.75–5.33)	0.17
Ever ran away as a child	25 (13.4)	9 (4.8)	3.00 (1.35–6.68)	0.007
Ever lived in a children's home	6 (3.1)	10 (5.2)	0.60 (0.22–1.65)	0.32
Abusive experiences in childhood^d^	31 (16.8)	33 (17.8)	0.93 (0.54–1.58)	0.79

^a ^Pair-wise analyses conducted on 194 matched case-control sets, except for: marital status (189/194 = 97.4%); no current relationship (184/194 = 94.8%); responsible for child < 18 yrs. (191/194 = 98.5%); not been out socially in last month (182/194 = 93.8%); practising a religion (192/194 = 99.0%); improved standard of living post-1990s (186/194 = 95.9%); born prematurely (155/194 = 79.9%); suicide by 1^st ^degree relative (189/194 = 97.4%); ever ran away as a child (187/194 = 96.4%); ever lived in a children's home (193/194 = 99.5%); abusive experiences in childhood (185/194 = 95.4%)

^b ^Reference category is married/cohabiting

^c ^Analysis of two explanatory variables based on smaller number of matched case-control sets, due to ineligibility according to age: 'ever been unemployed since 1990' (N = 130 sets); 'concerns over work prospects' (N = 122 sets)

^d ^Includes sexual, physical or emotional abuse during childhood

**Table 2:** Univariate conditional logistic regression models of clinical and recent major life event risk factors for suicide^a^

Risk factor	Cases n (%)	Controls n (%)	Odds Ratio (95% CI)	P-value
**Clinical factors**				
Lifetime history of psychiatric illness	83 (43.5)	29 (15.2)	4.38 (2.54–7.53)	< 0.001
Mental illness on reference date:^b^				
(i) Any diagnosis^c^	134 (69.1)	50 (25.8)	6.60 (3.83–11.36)	< 0.001
(ii) Affective disorder^d^	95 (49.0)	12 (6.2)	14.83 (6.49–33.90)	< 0.001
(iii) Dysthymia	17 (8.8)	10 (5.2)	1.78 (0.79–4.02)	0.17
(iv) Anxiety & related disorders	15 (7.7)	16 (8.2)	0.94 (0.46–1.90)	0.86
(v) Personality disorder	65 (33.5)	16 (8.2)	6.44 (3.19–13.01)	< 0.001
(vi) Alcohol/drug-related disorder^e^	57 (29.4)	20 (10.3)	3.64 (2.02–6.58)	< 0.001
Current smoker	103 (53.1)	50 (25.8)	3.52 (2.17–5.72)	< 0.001

**Recent life events**				
Interpersonal	67 (34.5)	38 (19.6)	2.26 (1.38–3.69)	= 0.001
Work or educational	39 (20.1)	33 (17.0)	1.22 (0.73–2.03)	0.44
Health (self or close relative)	42 (21.6)	11 (5.7)	6.17 (2.60–14.61)	< 0.001
Financial	28 (14.4)	20 (10.3)	1.50 (0.80–2.82)	0.21
Forensic/legal	33 (17.0)	8 (4.1)	4.57 (2.02–10.36)	< 0.001
Timing of life event:				
(i) Within last month	46 (23.7)	14 (7.2)	5.00 (2.34–10.68)	< 0.001
(ii) Within last 3 months	90 (46.4)	40 (20.6)	3.94 (2.31–6.71)	< 0.001
(iii) Within last 6 months	143 (73.7)	87 (44.8)	3.15 (2.03–4.90)	< 0.001

^a ^Pair-wise analyses conducted on all 194 matched case-control sets, except for: lifetime history of psychiatric illness (191/194 = 98.5%)

^b ^Measured using the Hungarian version of the Mini Neuropsychiatric Interview (M.I.N.I. v5.1) [11-13] to generate DSM-IV diagnoses

^c ^Includes affective disorder, dysthymia, schizophrenia & related disorders, manic episode, generalised anxiety disorder, hypomania, panic disorder, agoraphobia, social phobia, OCD, PTSD, alcohol abuse/dependence, drug abuse/dependence, eating disorders

^d ^Includes major depressive disorder and bipolar disorder

^e ^Abuse or dependence

**Table 3:** Multivariate conditional logistic regression model of suicide risk factors^a^

Risk factor	Adjusted OR (95% CI)	P-value
Current affective disorder	10.94 (3.84–31.12)	< 0.001
Alcohol/drug-related disorder	3.34 (1.28–8.76)	0.01
Current smoker	2.85 (1.21–6.74)	0.02
Single, separated, divorced or widowed	2.63 (1.03–6.70)	0.04
Not been out socially in last month	5.01 (2.23–11.25)	< 0.001
No education beyond age 16 yrs.	3.71 (1.07–12.81)	0.04
Major life event during last 3 months	4.29 (1.77–10.41)	0.001

^a ^Multivariate pair-wise analysis conducted on 91.8% (178/194) of all matched case-control sets

**Table 4:** Sensitivity analysis: univariate models with the data set restricted to 65 controls (and 65 matched cases) who did not act as their own informant^a^

Risk factor	Cases n (%)	Controls n (%)	Odds Ratio (95% CI)
Living alone	15 (23.1)	7 (10.8)	2.33 (0.90–6.07)
Responsible for child aged < 18 yrs.	11 (16.9)	10 (15.4)	1.14 (0.41–3.15)
Not been out socially in last month	29 (46.8)	6 (9.7)	6.75 (2.36–19.29)
Practising a religion	8 (12.3)	25 (38.5)	0.26 (0.11–0.64)
Born prematurely	6 (11.3)	11 (20.8)	0.55 (0.20–1.47)
Abusive experiences in childhood	9 (14.3)	12 (19.0)	0.73 (0.29–1.81)
Lifetime history of psychiatric illness	29 (44.6)	8 (12.3)	6.25 (2.18–17.96)
Any current psychiatric illness	45 (69.2)	17 (26.2)	6.60 (2.58–16.91)
Current alcohol/drug disorder	17 (26.2)	7 (10.8)	2.43 (1.01–5.86)
Current smoker	34 (52.3)	11 (16.9)	8.67 (2.62–28.63)
Interpersonal adverse life event	23 (35.4)	6 (9.2)	5.25 (1.80–15.29)
Work or educational adverse life event	13 (20.0)	10 (15.4)	1.33 (0.56–3.16)
Financial adverse life event	10 (15.4)	6 (9.2)	1.67 (0.61–4.59)
Adverse life event within 3 months	27 (41.5)	11 (16.9)	4.20 (1.58–11.14)
Adverse life event within 6 months	49 (75.4)	24 (36.9)	4.12 (1.91–8.93)

^a ^Analyses restricted to 65 matched case-control sets, with complete data except for: not been out socially in last month (62/65 = 95.4%); born prematurely (53/65 = 81.5%); abusive experiences in childhood (63/65 = 96.9%)

## Results

### Recruitment

During the study period informants for 262 individuals who had died by suicide were invited to take part in the study; 215 (82%) consented, and 194 (74%) were successfully interviewed. Forty-seven informants did not consent. The most common reason for refusal (in approximately 70% of cases) was that the relatives were too distressed to talk about the death. Other reasons for refusal included not wanting to speak ill of the deceased (suicide is associated with considerable stigma in Hungary) and not feeling it was their responsibility to talk about the reason their relative took his or her life. Twenty-one informants consented but changed their mind before the interview took place.

With respect to the controls, 233 individuals were approached, 216 (93%) consented, and 206 (88%) were successfully interviewed. Seventeen subjects did not consent for the following reasons: they did not want to talk about suicide; they did not want to talk to a psychiatrist. Ten individuals initially consented but were not interviewed because they subsequently decided that they did not wish to take part. The 206 interviews referred to 194 individual controls (12 control subjects had two informants). Where there was disagreement in the information obtained from informants, this was resolved by reference to coroners' and other files and discussion between the research team. For 129 subjects (66%) information was collected directly from the controls themselves. Third party informants were interviewed for 65 controls (34%).

### Description of informants

Of the 194 informants for the cases who were interviewed, 131 were female (68%). The median age of informants (IQR) was 48.5 years (36 to 55). With respect to the informants' relationship to the deceased, 50 (26%) were partners or spouses, 56 (29%) were parents, 45 (23%) were their children, 25 (13%) were siblings, 9 (5%) were other close relatives, and 9 (5%) were close friends or acquaintances. Thus, 91% of the informants were 1^st ^degree relatives and 95% were 1^st ^or 2^nd ^degree relatives. Almost half of informants lived with the deceased (92 informants, 48%). Of the remainder, 49 (26% of the total) had weekly or more frequent contact with the deceased, and a further 41 (21%) had at least monthly contact.

Third party informants were interviewed for 65 controls. Of these, 54 (83%) were female. The median age of these informants (IQR) was 46 years (33 to 64). Fifty-two informants (80%) were partners and 7 (11%) were parents. The majority lived with the controls (57 subjects, 88%).

### Description of cases

Of the 194 cases of suicide, 157 (81%) were male. The median age (IQR) was 43 years (30 to 62 years). The most common methods of suicide were hanging or strangulation (73 cases, 38%), drug poisoning (42 cases, 22%) jumping from high places (39 cases, 20%), and jumping in front of moving vehicles (16 cases, 8%). One hundred and eight subjects (57%) had directly or indirectly communicated an intent to die before their death and 58 (30%) had left a suicide note. The majority of deaths (124 cases, 64%) took place at the home of the deceased.

### Univariate models

There were differences between cases and controls in all domains (Tables 1 and 2). In the socio-demographic and other background domains (Table 1), strong and significant risk factors for suicide were being unmarried, having no current relationship, not having been out socially in the last month, lack of higher education, current unemployment or long-term sickness, having been unemployed at any time since 1990, concern over work prospects, suicide by a 1^st ^degree relative, and a history of having run away in childhood. The following factors were protective: non-white ethnicity, being responsible for a child, practising religion, and experiencing an improvement in living standards since 1990. Abusive experiences in childhood did not appear to be associated with risk of suicide.

In the clinical domain (Table 2), risk factors included current mental illness (particularly affective disorder – including bipolar disorder, but also personality disorder and alcohol/drug related disorders), psychiatric illness prior to the current episode, and being a current cigarette smoker. Twenty eight percent of cases had actually harmed themselves, and a further 10% had made threats of such behaviour; the prevalence of self-harm acts/threats among controls was very low (n = 3, 1.6%) and these sparse data precluded odds ratio estimation. We found no protective clinical factors. Although the rate of affective disorder among those who had died by suicide was 49% (95/194), the rate of antidepressant prescription among all cases was only 18% (35/194), and among the cases with affective disorder it was 20% (19/95).

Interpersonal, health-related and legal life events in adulthood (Table 2) were also important risk factors for suicide. Life events were associated with the greatest risk if they had occurred during the previous month, with a clear trend in falling odds ratio with greater length of time since occurrence of an adverse life event.

### Multivariate models

The independent risk factors for suicide identified in the multivariate model (Table 3) were current affective disorder, alcohol/drug-related disorder, current smoker, being single, separated, divorced or widowed, not gone out socially in the last month, no education beyond age 16 years, and recent occurrence of a major life event. The final model should be interpreted cautiously – one of the strongest univariate risk factors (previous self-harm) could not be included because of the small cell value for controls (n = 3). None of the protective factors identified in the univariate models were retained in the final multivariate model.

### Sensitivity analyses

Mixing measurements obtained directly from two thirds of the control subjects with proxy measurements given as third party accounts (for a third of the controls, and all of the cases) raised the likelihood of information bias. We therefore refitted the univariate models shown in Tables 1 and 2 by restricting the data set to the 65 controls who did not act as their own informant (and their 65 matched cases). The additional models included in the sensitivity analysis are shown in Table 4. In general the patterns of association were similar, with and without the restriction applied, although with the restricted data the observed odds ratios tended to be somewhat larger. This occurred because there was a generally lower level of reported exposure prevalence among the controls who did not act as their own informant.

To assess likelihood of reporting bias we compared the prevalence of variables reported in Tables 1 and 2, among the cases only, according to informant's gender, age ( < 50 vs. 50+ years) and relationship to deceased (1^st ^degree relative vs. other type). We found no striking patterns in the prevalence of characteristics by these informant groups by gender and age. The number of cases without 1^st ^degree relative informants was small (n = 18, 9%) and again there were few differences in characteristics according to type of informant. However, compared to cases with a 1^st ^degree relative informant those with other informants were significantly more likely to be living alone (67% vs. 24%; Fisher's exact P < 0.001) or to have had only basic education (44% vs. 22%; Fisher's exact P = 0.04).

## Discussion

### Main findings

We carried out a large controlled psychological autopsy and with respect to our hypotheses we found that a number of socio-demographic and clinical factors reported in Western settings were associated with suicide in Hungary. We also found that variables reflecting individuals' experiences since economic restructuring were associated with suicide. Risk factors included a history of unemployment since 1990 and current concerns over work prospects. Protective factors included practising religion and improvements in living standards. Many of these variables did not appear in the final multivariate models, possibly because their effects were mediated through traditional risk factors for suicide such as depressive illness, drug and alcohol misuse, and social isolation.

Previous psychological autopsy studies have identified clinical and behavioural variables associated with suicide. The most consistently reported risk factors include psychiatric disorder, self-harm, life events, lack of social support, alcohol and drug misuse [5,8,17-23], and in rural parts of Asia the widespread availability of lethal toxic agents such as pesticides [24,25]. We found that cigarette smoking was associated with suicide. Longitudinal analyses suggest that the association could be largely the result of confounding factors and that smoking is unlikely to be causally related to suicidal behaviour [26]. Other studies have found an association between smoking and suicide risk even with adjustment for potential confounders [27]. The mechanism underlying this association remains unclear and requires investigation in future studies. Comparatively few psychological autopsy studies have examined the role of protective factors. Fewer still have considered the wider context in which the studies take place. Studies carried out in Hungary have used coroners' records [28], and those that have used inteviews have tended to consider a more limited number of variables with a focus, for example, on psychiatric disorder [5] or recent personal life events [22].

Previous ecological and cohort studies have suggested that macroeconomic factors such as employment and deprivation may have an influence on the incidence of suicide [29-32], and one study showed a marked decrease in suicide rates in the former USSR during the period of political and socio-economic change associated with Perestroika [33]. However, such investigations have been weakened by a lack of detailed individually-based clinical data. Our study identified a number of factors related to recent social and economic restructuring that seemed to be important determinants of suicide risk at an individual level, although our study design did not allow us to establish a causal link with societal change. A recent controlled psychological study of suicide in Hong Kong among adults aged 30–49 also suggested that socio-economic factors had a significant impact on suicide risk [34].

### Methodological issues

We carried out a large, systematic, controlled study in a country which had experienced significant social changes in the recent past. The current investigation is, to our knowledge, the largest study of multiple risk factors for suicide to be carried out in Eastern Europe. We believe our results are relevant to other post-communist countries in the region. However, our findings need to be interpreted in the context of a number of methodological limitations.

The principal weakness of the psychological autopsy design is recall bias. Informants are of course not blind to outcome and there may be selected recall (or reporting) of aspects of the subject's life. Informants may also be unaware of certain factors (for example, drug and alcohol misuse, relationship difficulties). We attempted to minimise this bias by reference to coroners' files and general practice records. The use of matched control subjects is a key strength, but a majority of the control subjects did not nominate informants. This is a not uncommon problem with studies of this type [35-37] and may have introduced some potential information bias. For control subjects, if those who acted as their own informants were more likely to recall past exposures than third party informants, this would mean that the odds ratios for the risk factors for suicide in Tables 1 and 2 are actually conservative values – they are likely to be underestimates. Post-hoc analyses revealed that control subjects with and without third party informants were similar in terms of age and gender, but those with third party informants were less likely to be single (16% vs. 32%). However, the two groups reported similar levels of exposure to variables which might be most subject to information bias (for example, abusive experiences in childhood, mental disorder, treatment for mental disorder, current life problems). The sensitivity analysis presented in Table 4 generally gave similar (if somewhat larger) odds ratios with the data restricted to controls not acting as their own informants. That the effect sizes tended to be larger in the restricted data gives a further indication that the estimates derived from the full data are likely to be conservative.

We obtained a high response rate for both cases and controls, but it is possible that those individuals for whom we could not obtain information differed systematically from those included in the study. Our ascertainment methods for cases and controls may have been an additional source of bias. Ethical considerations meant that we were not permitted to collect any information for subjects whose informants did not consent to take part, so this selection bias is difficult to quantify. However, we were able to compare the cases to all individuals dying by suicide in Hungary in 2003 [3]. A similar proportion (77.1%) were males, and as in our study hanging was the most common method of suicide (62.9%), followed by poisoning (16.4%), and jumping from high places (6.1%). Hanging deaths seemed underrepresented in our sample and poisoning and jumping deaths overrepresented, but this may reflect differences in methods of suicide in Budapest compared to the rest of the country [5,28]. With respect to the controls, the rates of background mental illness, particularly affective disorder, were consistent with those reported for the Hungarian general population [38]. The low reported rate of previous self-harm among the controls was also consistent with Hungarian general population studies [39].

### Clinical and research implications

Suicide rates in Hungary have fallen consistently over the last 20 years [2]. Our study identifies a number of societal factors that may be important determinants of the suicide risk in individuals. These include employment, religious belief, and changes in socio-economic circumstances. On the basis of this study we are unable to determine whether these variables are causally related to suicide and therefore cannot be certain of the efficacy of society-wide interventions to reduce suicide rates. Such strategies may also be extremely challenging to implement [40]. It is possible, however, that improvements in socio-economic conditions may be associated with future reductions in suicide incidence in Hungary. Further ecological and clinically-based studies, not only in Hungary but in other countries in Eastern Europe, should be carried out to monitor rates of suicide and explore the possible relationship with societal factors.

The clinical risk factors we identified may indicate where further improvements in suicide rates will come from. Better recognition and treatment of psychiatric disorder, particularly depression, and effective management of those who self-harm may be the most productive clinically-based strategies to reduce suicide in Hungary [41]. In this study at least 95 cases (almost half of those who had died by suicide) had evidence of affective disoder at the time of death but only a fifth of these people had been receiving antidepressants. The situation is not that different from 20 years ago when a previous Hungarian psychological autopsy study found that just 15% of individuals with affective disorder who had died by suicide were taking antidepressants [5,42]. Although psychiatric services in Hungary have improved [2], there may still be considerable scope for further suicide prevention through the rigorous management of psychiatric disorder. Cohort studies or experimental designs could help to evaluate the effect of improved management of depression on future suicidal behaviour [43]. In general there has been an eight-fold increase in the rate of antidepressant prescription in Hunagry over the last two decades so one focus of investigation might be the *appropriate *prescription of antidepressants to those in clinical need. Almost thirty percent of cases had a lifetime history of non-fatal suicidal behaviour in this study, and a further ten percent had made threats of this sort. Previous work has sought to investigate the characteristics of those who self-harm in Hungary [13,44,45], but none have considered subsequent clinical management or self-harm as an outcome measure. Self-harm has received little attention in other Eastern European countries and future work should address this important topic.

## Conclusion

Similar to studies in Western settings, our study identified a number of risk factors related to individual-level demographic and clinical characteristics. We also identified a number of factors possibly related to recent economic and societal change. Suicide rates in Hungary have fallen considerably. Improved management of psychiatric disorder and self-harm may result in further reductions in suicide rates.

## Competing interests

Louis Appleby is National Director of Mental Health in England. The other authors have no competing interests to declare.

## Authors' contributions

LA, ZR, MK and GD took a leading role in the design of the study. All authors contributed to aspects of the study design. KA and NB carried out the data collection with supervision and advice from ZR, NK and JC. SH and RW carried out the statistical analysis with input from GD and NK. NK and ZR wrote the initial draft of the manuscript. All authors were involved in critically revising the initial draft. All authors read and approved the final manuscript.

## Pre-publication history

The pre-publication history for this paper can be accessed here:

http://www.biomedcentral.com/1471-244X/9/45/prepub
